# Effects of Carbonization Temperature and Component Ratio on Electromagnetic Interference Shielding Effectiveness of Woodceramics

**DOI:** 10.3390/ma9070540

**Published:** 2016-07-02

**Authors:** Yubo Tao, Peng Li, Sheldon Q. Shi

**Affiliations:** 1 College of Material Science and Engineering, Northeast Forestry University, Harbin 150040, China; tyblp@aliyun.com; 2 Department of Electrical Engineering, University of North Texas, Denton, TX 76203, USA; sheldon.shi@unt.edu

**Keywords:** woodceramics, electromagnetic interference, shielding effectiveness

## Abstract

Woodceramics were fabricated in a vacuum through carbonization of wood powder impregnated with phenol formaldehyde (PF) resin. The effects of carbonization temperature and mass ratio of wood/resin on electromagnetic interference (EMI) shielding effectiveness (SE) and morphology of woodceramics were explored. The PF resin made wood cell walls have the characteristics of glassy carbon. Wood axial tracheid and ray cells were filled with more glassy carbon by increasing addition of PF resin. Moreover, the increase of carbonization temperature was beneficial to improving SE. Woodceramics (mass ratio 1:1) obtained at 1000 °C presented a medium SE level between 30 MHz and 1.5 GHz.

## 1. Introduction

Electromagnetic interference (EMI) occurs when electronic devices are disturbed by unwanted electromagnetic radiation. EMI shielding refers to reflecting and/or absorbing electromagnetic radiation by a material that acts as a barrier. The importance of EMI shielding relates to demand for reliable electronics and rapid growth of radiation sources [1,2,3].

EMI shielding mechanisms include reflection, absorption, and multiple reflections. The primary mechanism, reflection, relies on mobile charge carriers (electrons or electron holes) present within the material. Therefore, electrical conductive materials, such as metals, are often used even though not typically required. However, shielding was found to be enhanced by connectivity [1,2]. The secondary mechanism is absorption. In order to achieve a significant absorption rate, the barrier should have electric and/or magnetic dipoles that interact with the electromagnetic radiation fields. Absorption loss is proportional to shield thickness and is a function of *σ_r_*, *μ_r_*, where *σ_r_* is the electrical conductivity relative to copper and *μ_r_* is the relative magnetic permeability. Absorption increases with increasing frequency whereas reflection tends to decrease [1,2]. The third mechanism is multiple reflections, which refers to reflections at various surfaces or interfaces in the shield. This mechanism requires the presence of large surface areas or interfaces within the shield. Examples of this kind of shielding are composite materials containing fillers with large surface areas. Thus, it is primarily based on the structural geometry properties of the composite materials. The loss due to multiple reflections can be neglected when the distance between the reflecting surfaces or interfaces is greater than the skin depth. The skin depth decreases with the increase of frequency, conductivity or permeability [1,2].

The performance of a shielding material is often defined by EMI shielding effectiveness (SE). This value, in decibels (dB), provides an indication of a material’s shielding quality. The medium SE for most shielding materials is 30 dB. This corresponds to a satisfying reduction of 99.9% of the interference signal [4]. Polymer composites and metallic materials are by far the most common candidates for EMI shielding materials [5]. A number of researches [6,7,8] focus on using polymer composites loaded with conductive fillers, fibers, nanotubes and/or dispersing particles. Those approaches, however, have drawbacks, namely: difficult processing, non-recyclability and high cost. As for metallic materials with excellent electrical conductivity or high magnetic permeability, such as copper, silver, and nickel, though exhibit great EMI shielding properties, high weight and density restrict some of their applications [9,10,11]. Therefore, developing a lightweight material with reasonable shielding effectiveness and enough mechanical properties is necessary.

Woodceramics are environmentally friendly and shapeable porous carbon materials made of wood or woody material impregnated with phenolic resin and carbonized in a vacuum at high temperatures. During the pyrolysis, the wood or woody material changes into soft amorphous carbon and the impregnated phenolic resin changes into hard glassy carbon. It is noteworthy that woodceramics inherit the hierarchic porous structure of wood. Hence, woodceramics are porous composite materials composed of amorphous carbon and glassy carbon [12]. Interesting applications of woodceramics have been found, such as thermal insulation, high-temperature filtration, and catalysis [13,14,15].

Interest has also arisen to develop woodceramics for EMI shielding. Shibata et al. [14] made woodceramics from waste paper with phenol resin in a thermal vacuum furnace. Results show that the wastepaper woodceramics have an electric shielding effectiveness of 30 dB at 100 MHz and 40 to 43 dB at 300 MHz or higher, and a magnetic shielding effectiveness of 30 dB at 100 MHz and 37 dB at about 400 MHz. It is proposed that the excellent SE is caused by dielectric loss [14]. Suda et al. [16] prepared woodceramics by burning medium density fiberboard (MDF, a non-structural wood fiber panel bound by adhesives under heat and pressure) impregnated with PF resin at 650 and 750 °C, then examined the properties under relative humidity 10%–70% and temperature from 20 to 100 °C. It was found that the woodceramics’ electrical resistance decreases linearly with the increase of temperature and/or relative humidity. With superior linearity between the electrical resistance and humidity/temperature [16], woodceramics can potentially be used for humidity and temperature sensors. Oh et al. [17] investigated electrical properties of pine sawdust woodceramics at different carbonizing temperatures and resin impregnation ratios. The electrical resistance decreases with the increase of carbonizing temperatures (no indication of further changes above 1000 °C) and/or the resin impregnation ratio. The findings suggested the potential utilization of woodceramics for EMI shielding, heating, and sensor materials [17]. Li et al. [18] prepared the woodceramics (carbonized tobacco stems and PF resin composite) by microwave irradiation. Results show that the mass loss ratio, volume shrinkage ratio and apparent density increase with the extension of microwave irradiation time, whereas the volume electrical resistivity and open porosity decrease. Similarly, as the apparent density increases with the mass fraction of PF resin, and the mass loss ratio, volume shrinkage ratio, volume electrical resistivity and open porosity decrease. The mass loss ratio, volume shrinkage ratio and open porosity increase with increase of microwave power, whereas the apparent density and volume electrical resistivity decrease [18].

Although some EMI shielding properties of woodceramics have been researched, little is known about the effects of processing parameters on the EMI SE of woodceramics. This study aimed at the fabrication of woodceramics by carbonizing wood powder impregnated with PF resin under high temperatures and vacuum circumstance. Effects of carbonization temperature and the ratio of wood/resin on EMI SE and microstructure of woodceramics are explored. The data obtained is expected to contribute to residual wood utilization as a high value byproduct of the wood processing industry.

## 2. Materials and Methods

### 2.1. Woodceramics Preparation

Woodceramics were prepared with laboratory-made Fir (*A. amabilis*) powder and laboratory synthesized PF resin. The Fir powder used has a moisture content of 8% and a mesh size <40 mesh and the PF resin has a solid content of 48%–50%, a viscosity of 30–40 s (TU—4 viscometer), an alkali content of 2.0%–2.7%, bromides of 16%–24%, and a storage period of at least 60 days at 5–20 °C. The fully PF resin impregnated pre-format wood powder was prefabricated in three steps, i.e., cold-pressing, drying, and hot-pressing with ratios of wood powder to resin 60:40, 50:50 and 40:60. Samples were then carbonized at temperatures 650, 800 and 1000 °C in a vacuum chamber. According to electromagnetic shielding equipment requirements, the specimens were prepared as disks with 110 ± 2 mm diameter and 7 mm of thickness.

### 2.2. Morphology

The morphology of the woodceramics samples was examined with a scanning electron microscope (SEM) (QUANTA200, FEI, Hillsboro, OR, USA). The samples were gold-coated before scanning. The accelerating voltage was 5 kV. The SEM images were obtained at different zones on each sample.

### 2.3. Measurement of EMI SE

**Figure 1:**
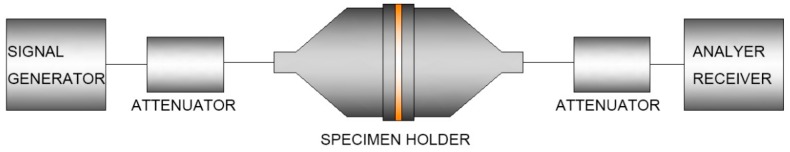
Schematic diagram of flange coaxial transmission line device for electromagnetic interference (EMI) shielding effectiveness (SE) test.

Based on the ASTM D 4935-99 “Standard Test Method for Measuring the Electromagnetic Shielding Effectiveness of Planar Materials”, the EMI SE measurement system was utilized (a flange coaxial transmission line testing device developed by the Southeast University, Nanjing, China). The measured frequency ranged from 30 MHz to 1.5 GHz. The experimental components and connection of the device are shown in Figure 1.

## 3. Results and Discussion

### 3.1. Morphology

**Figure 2:**
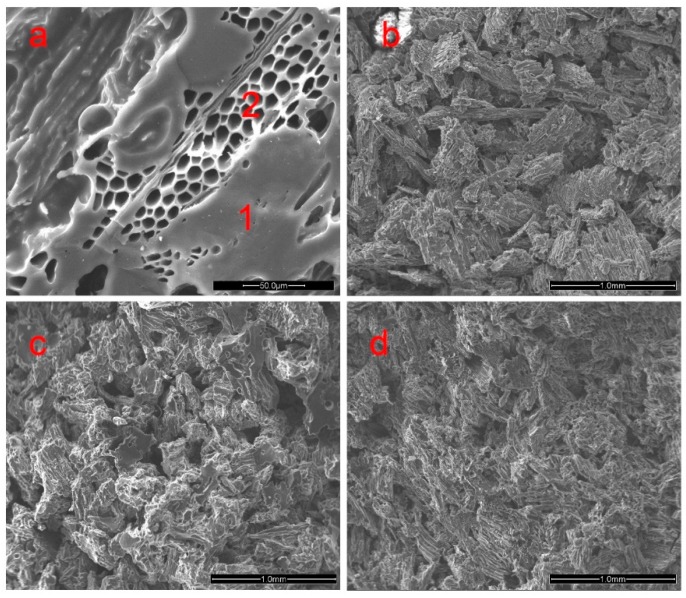
The microstructures of woodceramics under different carbonization temperatures (wood: resin = 40:60). (**a**) amorphous carbon and glassy carbons; (**b**) 650 °C; (**c**) 800 °C; and (**d**) 1000 °C.

Figure 2a shows that woodceramics are typically porous carbon materials, furthermore, the portion pointed by number 1 displays glassy carbon generated by PF resin, the portion pointed by number 2 shows amorphous carbon formed by wood powder, while black areas demonstrate voids. This kind of microstructure is in consistent with the findings from Zhang et al. and Qian et al. [19,20]. Figure 2b–d present SEM images of woodceramics at carbonization temperatures of 650, 800 and 1000 °C, respectively. Compared to Figure 2c,d, the structure on the cross-section was loose and the continuous phase was rarely observed when woodceramics were sintered at 650 °C (Figure 2b). However, the glassy carbon continuous phase can be clearly observed on the woodceramics sintered at 800 °C (Figure 2c). In contrast, the continuous phase gradually reduced as the sintering temperature reached 1000 °C. This phenomenon was probably caused by the gasification of glassy carbon being stronger at 1000 °C. The impregnated wood powder shrank and carbon particle contacted closely with the rising carbonization temperature. The distance between carbonized wood powders was enlarged as temperature increased due to carbonized wood powder’s greater shrinkage rate.

**Figure 3:**
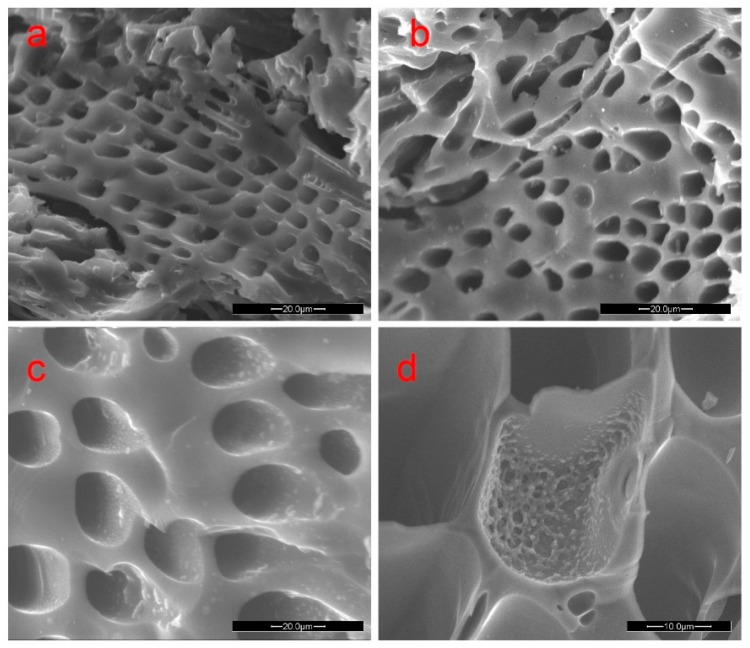
SEM images of different wood/resin ratios at 1000 °C. (**a**) 60 (wood):40 (resin); (**b**) 50 (wood):50 (resin); (**c**) 40 (wood):60 (resin); and (**d**) glassy carbon left by carbonization PF in void.

Figure 3a–c show microstructures of the woodceramics at 1000 °C with different wood/resin ratios, 60:40, 50:50 and 40:60, respectively. As seen in the images, all wood cell walls were enforced through filling PF resin. With the major wood structure remaining in good shape, wood cell walls began showing the characteristics of glassy carbon after the carbonization and wood structures became denser resulting in the disappearance of wood pits. Figure 3d shows that phenolic resin can also fill in the voids of wood. Zhang et al. [19] also found that certain amounts of phenolic resin are able to block the porous structures of carbonized tobacco stems. As the content of PF resin increased, glassy carbon left from the carbonization of PF resin was observed in wood axial tracheid and ray cells. The glassy carbonization phenomenon was more noticeable at 40:60 wood/resin ratio.

### 3.2. Effect of Carbonization Temperature on EMI SE

**Figure 4:**
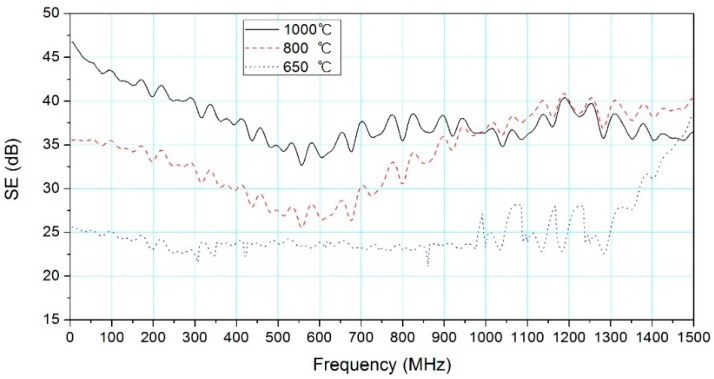
Effect of carbonization temperature on SE (wood:resin = 50:50, apparent density of 0.649 g/cm^3^ (650 °C), 0.645 g/cm^3^ (800 °C) and 0.639 g/cm^3^ (1000 °C) respectively).

As shown in Figure 4, SE of woodceramics (1000 °C) decreased sharply from 47 to 33 dB with the increase of frequency from 30 to 550 MHz, and SE fluctuated between 35 and 40 dB in the bandwidth of 550 MHz–1.5 GHz. It is clear that all SEs were in excess of 30 dB from 30 MHz to 1.5 GHz, which indicated that the woodceramics presented a medium EMI SE level.

SEs (800 °C) were mostly over 30 dB in the tested frequency range. SE decreased from 35 to 26 dB (30 MHz–550 MHz) and then increased gradually to 40 dB (550 MHz–1.5 GHz). Medium EMI SE level is achieved with frequencies over 750 MHz.

The range of SEs (650 °C) is mostly around 25 dB. SE increased rapidly with frequencies above 1.3 GHz. When the frequencies were over 1.35 GHz, SEs were greater than 30 dB.

The effects of carbonization temperature on SE vary from frequency ranges. SE improved at higher carbonization temperatures in the frequency range of 30 MHz–1.0 GHz. According to the primary mechanism for EMI shielding, high electrical conductivity could enhance SE. The study of Oh et al. [17] showed that the electrical resistance can be decreased with the increase of carbonization temperature. Therefore, lower electrical resistance is beneficial to improving electrical conductivity and SE of woodceramics. According to the multiple reflections mechanism, this attenuation requires the presence of large surface areas or interface areas as in a porous or foam material, or a composite material containing a filler which has a large surface area in the shield [1]. SEM images showed that when the woodceramics were sintered from 800 °C to 1000 °C, the size of carbonized continuous phase, wood powder and carbon-particle contact areas were gradually reduced, resulting in the increase of surface area. Chung et al. found that, due to the skin effect (i.e., the phenomenon that high-frequency electromagnetic radiation only interacts with the surface region of a conductor), larger surface area should provide better shielding [1].

In the frequencies of 1.0–1.45 GHz, the SE of woodceramics obtained at 800 °C was slightly higher than those at 1000 °C. SE at 650 °C rapidly increased with frequencies above 1.3 GHz. This trend could be explained by dielectric loss. Larger permittivity can be obtained from the dielectric function measurement of the woodceramics in a higher frequency [14]. Therefore, it is proposed that the dielectric loss of woodceramics leads to an increase of EMI SE.

The structure of woodceramics can cause reflection, absorption, and multiple reflections, which could make SE obtained at 800 °C greater than that obtained at 1000 °C above 1.0 GHz. SEs obtained at 800 °C and 1000 °C were both greater than that obtained at 650 °C. Therefore, the increase in carbonization temperature can enhance SE.

### 3.3. Effect of Wood/Resin Ratio on EMI SE

**Figure 5:**
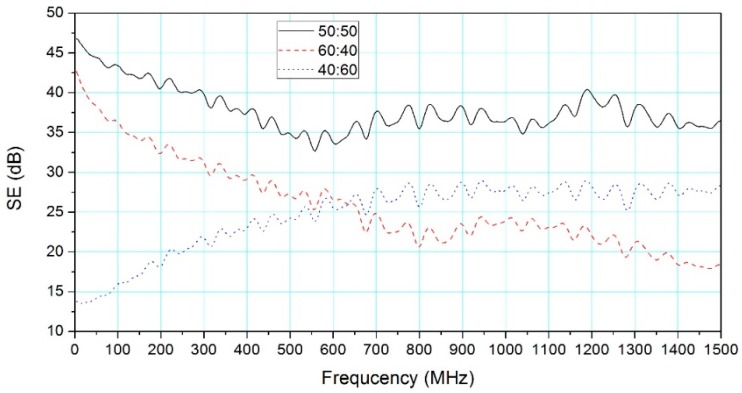
Effect of wood/resin ratio on SE (carbonization temperature of 1000 °C, apparent density of 0.639 g/cm^3^ (50:50), 0.625 g/cm^3^ (60:40) and 0.642 g/cm^3^ (40:60), respectively).

Figure 5 demonstrated the influences of wood/PF resin ratio on SE of woodceramics in the frequency range from 30 MHz to 1.5 GHz. SE obtained with the wood/PF resin ratio of 50:50 was the highest among all tested ratios. When the wood/resin ratio was 60:40, SE gradually reduced with rising frequency. In the lower frequency range of 30 MHz–350 MHz, SEs were over 30 dB. In contrast, when the wood/PF resin ratio was 40:60, SE increased steadily between 30 and 700 MHz and then fluctuated around 27 dB from 700 MHz to 1.5 GHz.

According to the study of Shibata et al. [14], the carbonization temperature and PF resin content are directly related to the specific surface area of the woodceramics [14,21]. The study of Li et al. [18] also showed that the greater the fraction of PF resin, the greater the apparent density of woodceramics. The mass loss ratio, volume shrinkage ratio, volume electrical resistivity and open porosity decrease with the quantity increase of PF resin [18]. This could be explained by the proposition that the resin content influenced the woodceramics SE. Therefore, SE of woodceramics depends on the quantity of PF resin impregnated.

## 4. Conclusions

The following conclusions were drawn from this study:

As the carbonization temperature increased, the continuous phase glassy carbon in woodceramics increased then decreased. The role of PF resin was to carbonize wood cell walls for the characteristics of glassy carbon. An increase in resin content can deposit glassy carbon into the wood axial tracheid and ray cells.

At carbonization temperatures of 650, 800 and 1000 °C, and wood/resin ratio of 50:50, the woodceramics had EMI SE in the frequency range of 30 MHz–1.5 GHz. An increase in carbonization temperature enhanced the SE. As for the carbonization temperature of 1000 °C, the SE of the woodceramics reached medium level.

The SE of the woodceramics was significantly affected by the wood/resin ratio. The SE of ratio 50:50 was considerably higher than those of ratios 60:40 and 40:60 in the frequency range of 30 MHz–1.5 GHz.
